# Current Views on Genetics and Epigenetics of Cholesterol Gallstone Disease

**DOI:** 10.1155/2013/298421

**Published:** 2013-04-14

**Authors:** Agostino Di Ciaula, David Q.-H. Wang, Leonilde Bonfrate, Piero Portincasa

**Affiliations:** ^1^Division of Internal Medicine Hospital of Bisceglie, 76011 Bisceglie, Italy; ^2^Saint Louis University School of Medicine, Division of Gastroenterology and Hepatology, Department of Internal Medicine, Edward Doisy Research Center, St. Louis, MO 63104, USA; ^3^Clinica Medica “A. Murri”, Department of Biomedical Sciences & Human Oncology, University “Aldo Moro“ of Bari Medical School, 70124 Bari, Italy; ^4^European Society for Clinical Investigation (ESCI), 3584 CJ Utrecht, The Netherlands

## Abstract

Cholesterol gallstone disease, one of the commonest digestive diseases in western countries, is induced by an imbalance in cholesterol metabolism, which involves intestinal absorption, hepatic biosynthesis, and biliary output of cholesterol, and its conversion to bile acids. Several components of the metabolic syndrome (e.g., obesity, type 2 diabetes, dyslipidemia, and hyperinsulinemia) are also well-known risk factors for gallstones, suggesting the existence of interplay between common pathophysiological pathways influenced by insulin resistance, genetic, epigenetic, and environmental factors. Cholesterol gallstones may be enhanced, at least in part, by the abnormal expression of a set of the genes that affect cholesterol homeostasis and lead to insulin resistance. Additionally, epigenetic mechanisms (mainly DNA methylation, histone acetylation/deacetylation, and noncoding microRNAs) may modify gene expression in the absence of an altered DNA sequence, in response to different lithogenic environmental stimuli, such as diet, lifestyle, pollutants, also occurring *in utero* before birth. In this review, we will comment on various steps of the pathogenesis of cholesterol gallstones and interaction between environmental and genetic factors. The epigenomic approach may offer new options for therapy of gallstones and better possibilities for primary prevention in subjects at risk.

## 1. Introduction

Cholesterol gallstone disease is one of the most prevalent and most costly digestive diseases requiring hospital admission, since its prevalence ranges from 10% to 15% in adults. Medical expenses for gallstone treatment exceeded $4 billion in facility charges in 2004 in the United States [1] and rise to $6.5 billion when surgical complications occur [2]. The formation and growth of cholesterol gallstones, which accounts for 75% of the gallstones in westernized countries [3–5], are secondary to abnormal cholesterol homeostasis [6]. Of note, the main risk factors for cholesterol gallstone disease (e.g., obesity, type 2 diabetes, dyslipidemia, and hyperinsulinemia) are also well-known components of the metabolic syndrome [7–11], supporting the hypothesis that gallstone disease is just another component of the metabolic syndrome [12–14] (Table 1). Due to the high prevalence of the metabolic syndrome, it has been suggested that the phenotype of cholesterol gallstones may result from the interaction between insulin resistance, genetic factors, and a number of environmental factors [15]. A series of gallstone (*LITH*) genes have been identified, which affect cholesterol homeostasis and promote cholesterol gallstone formation and growth [15]. Also, a strong interest has developed to investigate the epigenetic mechanisms that are able to influence gene expression in the absence of an altered DNA sequence [16], in response to several environmental stimuli [15].

A comprehensive analysis of these latter aspects as key factors in linking cholesterol homeostasis to gene expression and to the environment might provide a clue for both the prevention of gallstone formation in subjects at risk and future therapeutic approaches via manipulation of cholesterol homeostasis.

## 2. Cholesterol Homeostasis and the Formation of Cholesterol Gallstones 

### 2.1. Multifactorial Contributions to the Pathogenesis of Gallstones

**Figure 1:**
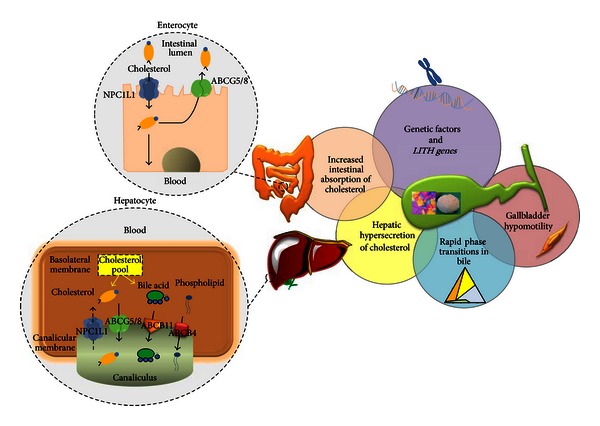
Current view on the complex interplay of pathogenic factors in cholesterol gallstone formation. The combination of multiple disturbances affecting cholesterol homeostasis in bile is essential for cholesterol gallstone formation. *LITH* genes and genetic defects play a crucial role in the formation of cholesterol gallstones. A large number of *LITH* genes have been identified in mouse models of cholesterol gallstones, and based on mouse studies, several human *LITH* genes have been identified, and their contributions to the formation of cholesterol gallstones are now being investigated. Hepatic hypersecretion of biliary cholesterol leads to unphysiological supersaturation of gallbladder bile with cholesterol. At the enterocyte (small intestine) level, absorption of cholesterol is enhanced via the Niemann-Pick C1-like 1 (NPC1L1) pathway. In bile, as a consequence, accelerated phase transitions of cholesterol occur, which are facilitated by prolonged gallbladder stasis due to impaired gallbladder motility and immune-mediated gallbladder inflammation, as well as hypersecretion of mucins and accumulation of mucin gel in the gallbladder lumen [6, 17]. In bile, growth of solid plate-like cholesterol monohydrate crystals to form gallstones is a consequence of persistent hepatic hypersecretion of biliary cholesterol together with enhanced gallbladder mucin secretion and incomplete evacuation by the gallbladder due to its impaired motility function [6, 29]. The two inlets on the left depict the major pathways of cholesterol absorption and secretion at the enterocyte level and at the hepatocyte level, respectively, as mediated by specific transporter proteins. Also, relative cholesterol hypersecretion into hepatic bile may or may not be accompanied by normal, high, or low secretion rates of biliary bile acids or phospholipids. Although NPC1L1 is expressed in the liver, its mRNA expression and protein concentrations are very low compared to those in the small intestine, thereby suggesting that hepatic NPC1L1 could have a minor role in regulating biliary cholesterol secretion.

Precipitation of excess cholesterol in bile as solid plate-like monohydrate crystals is a prerequisite for the formation of cholesterol gallstones [18, 19]. It is evident that all factors contributing to cholesterol homeostasis (i.e., intestinal cholesterol absorption, hepatic cholesterol biosynthesis, biliary output, and cholesterol conversion to bile acids) play a vital role in the pathogenesis of cholesterol gallstones. In fact, specific pathogenic factors concurring to the formation of cholesterol gallstones in humans must include hepatic hypersecretion of cholesterol into bile leading to a supersaturated bile, accelerated cholesterol nucleation/crystallization, defective gallbladder motility (a form of cholesterol-induced leiomyopathy leading to gallbladder stasis [6, 20, 21]), increased absorption of intestinal cholesterol, and *LITH* gene expression [17, 22–28] (Figure 1).

### 2.2. Liver, Bile, Intestine, Gene Expression, and Cholesterol Homeostasis

The liver plays a central role in cholesterol homeostasis and lipoprotein metabolism since it is mainly involved in synthesis and catabolism of cholesterol and lipoproteins and is the exclusive excretory route for cholesterol from the body [21]. 

In normal subjects with an extremely low dietary cholesterol intake (*∼*30 mg/day, pure vegetarians), biliary cholesterol mainly derives from *de novo* synthesis [33]. In the physiological steady state, hepatic secretion of biliary cholesterol principally derives from newly synthesized cholesterol, plasma lipoproteins (the main source of biliary cholesterol is HDL cholesterol, as mainly suggested by animal models [34–37]), and intestinal absorption of cholesterol. Dietary and reabsorbed biliary cholesterol is delivered by the enterolymphatic circulation to the liver for resecretion into bile. As demonstrated by both human and animal studies, reabsorption of biliary cholesterol by the enterocytes has different absorption efficiency [38, 39] and depends on sterol transport proteins compared to dietary cholesterol [40–42]. Intestinal absorption of cholesterol is a multistep process regulated by multiple genes [41], which is determined by the balance between influx and efflux of intraluminal cholesterol molecules crossing the brush border membrane of the enterocyte [41]. 

The rate of whole-body cholesterol synthesis by the liver is approximately 8–10 mg/day/kg body weight in humans [43], and, under normal physiological conditions, *de novo *synthesis contributes to biliary cholesterol secretion approximately by 15% [44–47]. Interestingly, cholesterol synthesis by the liver is suppressed by a negative feedback regulatory mechanism through the sterol regulatory elementary binding protein-1 (SREBP-1) pathway when dietary cholesterol intake is increased, which also induces an enhanced secretion of cholesterol into bile, the conversion of cholesterol into bile acids (subsequently for secretion into bile), an increased cholesterol esterification and storage, and an enhanced lipoprotein secretion into the circulation [21]. In humans, the fibroblast growth factor receptor 4 (FGFR4) may have an effect on maintaining bile acid homeostasis by regulating the expression of cholesterol 7alpha-hydroxylase (CYP7A1), the rate-limiting enzyme for the classic pathway of bile acid biosynthesis [48]. Additionally, the liver X receptor (LXR) plays a main role in cholesterol homeostasis because it can activate the transcription of the genes, such as ABCG5/8, ABCA1, and ABCG1, involved in the response to excess cholesterol intake [49–51]. In mice, it has been reported that there is an increased propensity to cholesterol crystallization and gallstone formation in bile following the activation of hepatic LXR and direct upregulation of the major cholesterol efflux transporters ABCG5 and ABCG8 on the canalicular membrane of hepatocyte [52]. 

### 2.3. Altered Cholesterol Homeostasis: The Lithogenic State

**Figure 2:**
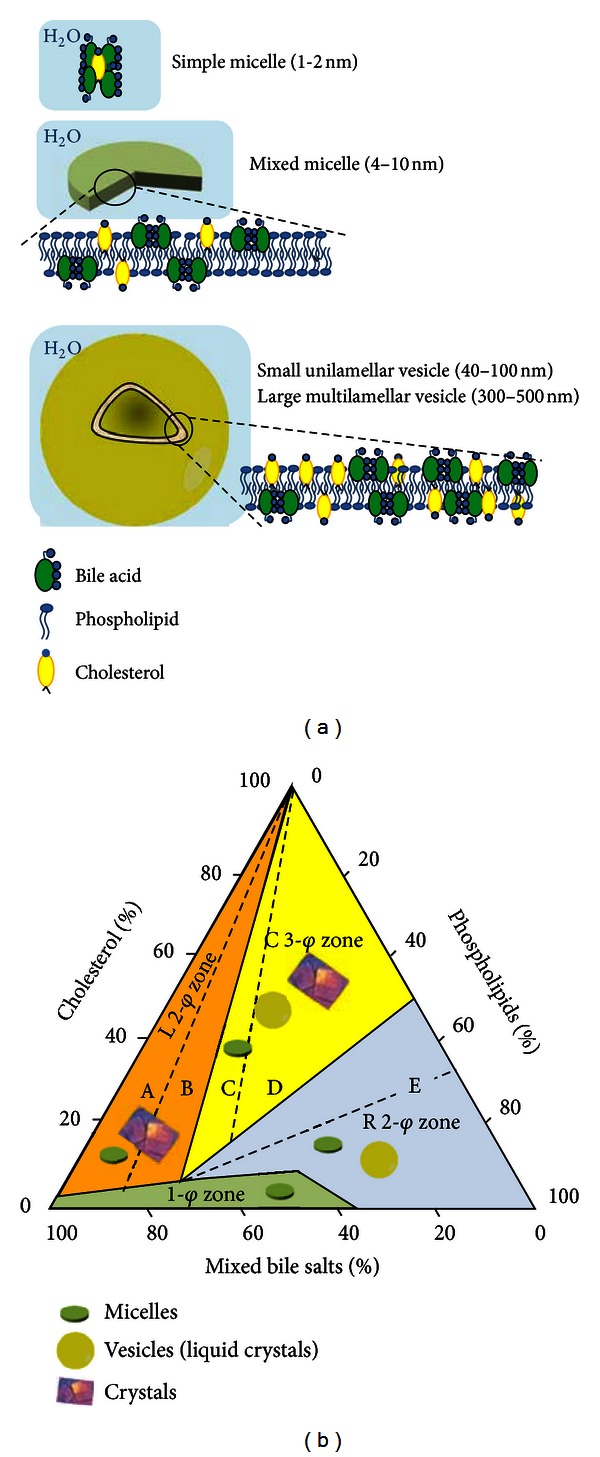
(a) The figure shows the physical states of lipids in human bile [21]. Bile is composed mainly of water (more than 90%) [30]. Bile acids are highly soluble, while cholesterol and phospholipids are highly insoluble in water. In bile, bile acids are found as monomers up to the critical micellar concentration (*≈*1–3 mM), a cut-off value after which bile acids can self-aggregate as simple micelles, binding a molecule of cholesterol. This step leads to increased aqueous solubility of cholesterol. Phospholipids in an aqueous environment can self-aggregate to form stable bilayer vesicles containing also a trace amount of bile acids, if any. A large amount of the cholesterol molecules is inserted into these bilayers of vesicles between the hydrophobic acyl chains of phospholipids. With typical gallbladder lipid concentrations and compositions, simple bile acid and mixed bile acid-lecithin micelles coexist in a ratio of 1 : 5. Unilamellar vesicles are larger spherical carriers in which even more cholesterol is solubilized into the bilayers of phospholipids. The ratio of unilamellar vesicles to micelles depends on the bile acid and phospholipid concentrations of bile, which is the greatest in bile with low bile acid and high phospholipid concentrations. Furthermore, at low bile acid concentrations and high phospholipid concentrations, these biliary phospholipids often form large multilamellar layers of vesicles. High concentrations of bile acids can dissolve these vesicles to form mixed micelles. (b) The picture depicts the ternary bile salt-cholesterol-phospholipid phase diagram in which the different pathways of cholesterol solubilization and/or precipitation in bile are shown [17]. The concentrations of three biliary lipids (bile acids, cholesterol, and phospholipids) are shown as percentages on the three axes of the triangle with a total lipid concentration of 7.2 g/dL, pH 7, and a temperature of 37°C [31, 32]. Different zones occupying areas within the triangle are shown, with each one containing different cholesterol carriers. The one-phase (*φ*) zone under the saturation curve contains only micelles and represents the bile being unsaturated with cholesterol. Above, three other zones exist with cholesterol supersaturation: a right two-phase (R 2-*φ*) zone containing saturated micelles and vesicles; a central three-phase (C 3-*φ*) zone containing saturated micelles, vesicles, and solid cholesterol crystals; and a left two-phase (L 2-*φ*) zone containing saturated micelles and solid cholesterol crystals. Whereas cholesterol precipitation is rapid in case of excess bile acids, at increasing amounts of phospholipids, cholesterol may reside in vesicles with phospholipids. At this moment, solid cholesterol crystal formation is slower or absent. Cholesterol crystallization, the first key step in cholesterol gallstone disease, is increasing at increasing concentrations of cholesterol, in the central and left zones, above the “safe” and physiological micellar zone.

Pathologic conditions linked to cholesterol gallstone formation in humans are characterized by a “lithogenic state,” in which the *de novo *synthesis could provide the liver with more cholesterol for secretion into bile. The current view on the physical chemistry of cholesterol carriers in bile is summarized in Figure 2. Bile contains the three classes of biliary lipids (i.e., bile acids, phospholipids, and cholesterol), and specific cholesterol carriers in health include simple and mixed micelle and small and large vesicles. Sustained cholesterol supersaturation in bile will lead to a cascade of events in which excess cholesterol will lead to nucleation and crystallization and finally precipitate as solid plate-like monohydrate crystals, the first key step in cholesterol gallstone formation.

Bile becomes desaturated with cholesterol after long-term administration of statins, the competitive inhibitors of HMG-CoA reductase, and the rate-limiting enzyme in cholesterol biosynthesis [53–60].

Compared to gallstone-resistant AKR mice, susceptible C57L mice on the lithogenic diet still display higher HMG-CoA reductase activities together with lower activities of both bile acid synthetic enzymes cholesterol 7*α*-hydroxylase and sterol 27-hydroxylase [61]. Furthermore, higher HMG-CoA reductase activities have been found in gallstone patients compared with control subjects [62–65]. This evidence underscores the role of *de novo* cholesterol synthesis in the formation of lithogenic bile in humans at risk for gallstones. 

The small intestine also plays a key role in the absorption of both dietary and biliary cholesterol, which is present in bile solely in the unesterified form (at least 97% of total sterols in bile) [15].

The importance of the gallbladder on the regulation of reabsorption of biliary cholesterol has been underlined by an animal model showing that the gallbladder can modulate the physical states of cholesterol, which may in turn influence the intestinal absorption of biliary cholesterol. In this model, crystallized bile markedly reduced cholesterol uptake and absorption by the enterocyte [42].

The average intake of cholesterol in the western diet is approximately 300–500 mg per day (predominantly animal origin). The small intestine contains both unesterified and esterified cholesterol, with the latter usually in small proportion [66]. Any cholesteryl ester entering the intestine must be hydrolyzed by pancreatic cholesterol esterase in order to be absorbed. Bile delivers 500–2400 mg of cholesterol per day to the intestine [67], and this amount is approximately two to three times the dietary cholesterol. An additional source of intraluminal cholesterol (about 300 mg of cholesterol per day) comes from the turnover of intestinal mucosal epithelium [41]. It has been demonstrated that intestinal factors have a major role in the pathogenesis of mouse gallstone formation [68], since the deficiency of cholesterol esterification in the intestine of ACAT2 knockout mice leads to a marked reduction in intestinal cholesterol absorption and complete resistance to diet-induced cholesterol gallstones.

Additionally, the lack of expression of intestinal Apo-B48 (but not Apo-B100) leads to a significant reduction in biliary cholesterol secretion and gallstone formation, possibly by decreasing intestinal absorption and hepatic bioavailability of cholesterol [69]. Apo-E knockout mice on the lithogenic diet show reduced biliary cholesterol secretion and gallstone prevalence, possibly due to a decreased availability of chylomicron-derived cholesterol in the liver for biliary cholesterol secretion [70]. Although results from animal studies underscore the role of cholesterol derived from the intestine on biliary cholesterol secretion and provide clear evidence that high dietary cholesterol through this pathway enhances cholelithogenesis, human studies concerning this topic gave conflicting results. It has been demonstrated that biliary cholesterol saturation increases with increasing cholesterol intake, inducing the formation of lithogenic bile and solid cholesterol crystals in some subjects [71]. Conversely, a group of 9 healthy women showed no increase in biliary cholesterol saturation after a high-cholesterol diet [72], and 6 normolipidemic women and 6 hyperlipidemic patients without gallstones showed no change in biliary cholesterol saturation when dietary cholesterol was increased from 300 mg to 1,500 mg daily [73]. An increase in biliary cholesterol saturation with modest increments in dietary cholesterol has been noticed in a group of 12 patients with asymptomatic gallstones (six men and six women), as compared with 7 healthy women (the diets containing 500, 750, and 1,000 mg of cholesterol daily for 3-week periods in random sequence). Interestingly, the biliary cholesterol saturation increased in this study group independently from the presence of gallstones [74]. Results from this study showed that women with gallstones had higher biliary cholesterol saturation than normal women at corresponding levels of cholesterol consumption, and six of the seven normal women formed lithogenic bile when ingesting a diet containing 1,000 mg of cholesterol. The discrepancies in human studies might be explained, at least in part, by differences in population sampling, by dissimilar diets, and by variations in the absorption efficiency of intestinal cholesterol. 

## 3. Genetic, Epigenetic, and Environmental Factors

The analysis of the mechanisms linking environmental factors to the genes in the determination of human health is of importance in the field of life sciences and biomedical research. Several studies have demonstrated that family history, genetics, dietary, and cultural habits have a main role in the onset of gallstones [75–77]. Furthermore, a number of observations have found that a complex genetic basis could play a key role in determining individual predisposition to develop cholesterol gallstones in response to environmental factors [22, 23, 26, 28], and a wide spectrum of environmental and genetic risk factors may influence the onset of gallstone disease in humans [78, 79]. The analysis of twin pairs from The Swedish Twin Registry showed that genetic factors are estimated to account for about 25% of gallstone risk [80] and that twins carrying a heterozygous or homozygous ABCG8 D19H genotype have a significantly increased risk of gallstone disease [81]. The *ABCG8 *p.D19H may lead to lower intestinal cholesterol absorption, lower serum cholesterol levels, and higher hepatic cholesterol synthesis, and polymorphisms in the ABCG5/ABCG8 genes are certainly related to the variations in plasma lipid levels, cholesterol saturation of bile [82], and insulin resistance [83]. An inventory of human cholesterol gallstone (*LITH*) genes has been depicted [15], and this list is rapidly growing. It has been recently suggested that susceptibility to gallstone disease may be influenced in humans by mucin gene polymorphisms [84] or FGFR4 polymorphism [48] and that the mucin-like protocadherin gene (MUPCDH) polymorphism rs3758650 has been considered a genetic marker to predict symptomatic gallstone disease [85]. Furthermore, carriers of CG genotype of ABCG8 rs11887534 showed higher risk of gallstones, as well as gallbladder and bile duct cancer compared with carriers of the GG genotype [86].

On the other hand, besides genes, the role of epigenetics has been highlighted by a number of human studies as the key factor in the onset of several chronic metabolic [87–92] and nonmetabolic diseases, such as cancer [93–95] cardiovascular diseases [96], neurodegenerative diseases [97], and birth defects [98], as a consequence of exposure to “toxic” agents occurring *in utero *before birth [16, 99]. They occur when the function of a gene is altered by various mechanisms, although its DNA sequence remains stable [16]. Transgenerational effects and fetal programming result from a mother's exposure and are inherited through successive generations in the absence of direct exposure of the offspring. Fetal programming, in turn, results in the onset of diseases in adult age, underlying the importance of developmental factors in influencing the risk of later-life disease [100]. Diet [101, 102] or environmental exposition to a number of chemical agents like heavy metals (e.g., cadmium, arsenic, nickel, chromium, and methylmercury) [103–107], air pollutants (e.g., particulate matter, black carbon, and benzene), and endocrine-disrupting/reproductive toxicants (e.g., diethylstilbestrol, bisphenol A, persistent organic pollutants, dioxin, and pesticides [108–112]) are able to induce epigenetic changes (mainly DNA methylation, histone acetylation/deacetylation [113], and noncoding microRNAs) [114, 115], which are involved in a wide range of metabolic diseases including obesity [90, 116], abnormal hepatic triglyceride accumulation [91], and the metabolic syndrome [92, 117], type 2 diabetes [87–89], all well-known risk factors for gallstone disease and mainly attributable to insulin resistance. Interestingly, it has been recently reported by a cluster of analyses a significant association of gallbladder diseases with environmental pollutants (heavy metals) in drinking water [118].

The interaction of histone acetyltransferases (HATs) and histone deacetylases (HDACs) and histones strongly affects gene transcription, and, in particular, it has been suggested that HDACs are important in the regulation of lipid homeostasis [113]. Of note, microRNAs (miR-122, miR-370, and miR-33) have a major influence on cholesterol homeostasis. They are important posttranscriptional regulators of gene expression [119–121] and strongly affect cholesterol metabolism [122]. It has been recently reported by an animal model that maternal low-protein diet during gestation and lactation significantly alters cholesterol homeostasis in weaning piglets through altered epigenetic regulation (promoter hypomethylation, decreased histone H3, H3 lysine 9 monomethylation, H3 lysine 27 trimethylation, and increased H3 acetylation) of the *HMGCR *(the rate-limiting enzyme in cholesterol biosynthesis) and *CYP7A1 *(the rate-limiting enzyme for conversion of cholesterol to bile acids) genes, with possible long-term consequences in cholesterol homeostasis later in adult life [101]. In the rat, maternal undernutrition leads to long-term dysregulation of cholesterol metabolism in the offspring through epigenetic mechanisms [102].

In humans, it has been reported that placental insufficiency-induced intrauterine growth restriction secondary to adverse events *in utero* may be responsible for metabolic events leading to the metabolic syndrome [102, 123, 124]. 

The bile acid receptor farnesoid X receptor (FXR) is currently considered to be the intracellular “sensor” of bile acids [125, 126]. Cells synthesize oxysterols under conditions of cholesterol overload, and oxysterols in turn bind and activate LXR, which acts to reduce systemic cholesterol burden [125–127]. FXR is highly expressed in the enterohepatic system and regulates the expression of the genes involved in the maintenance of cholesterol, bile acid, and triglyceride homeostasis [128]. Of note, it has been recently suggested by a comparison of genomic FXR-binding sites in healthy control and obese mice that FXR transcriptional signaling is altered in diet-induced obese mice, which may underlie aberrant metabolism and liver function in obesity [129]. 

In conclusion, frequent metabolic abnormalities such as atherosclerosis, obesity, metabolic syndrome, and gallstone disease are related to impaired cholesterol homeostasis. The current view that such abnormalities gain clinical relevance only during adulthood and elderly age is dramatically changing. Both genetic and epigenetic studies suggest a very early onset of chronic disease already *in utero*. Epigenetic mechanisms underlying such developmental events are still under investigation, in particular in the case of cholesterol homeostasis and gallstone disease. Starting from these particular metabolic conditions, a better understanding of mechanisms resulting in chromatin remodeling in response to environmental stimuli acting on the epigenome may offer new options for therapy of cholesterol cholelithiasis and better possibilities for primary prevention in subjects at risk.

## Figures and Tables

**Table 1:** Major risk factors for cholesterol gallstones.

Independent
(i) Increasing age
(ii) Female gender
(iii) Race
(iv) Family history
Dietary
(i) High calorie
(ii) High cholesterol
(iii) High fat
(iv) High *trans*-fatty acids
(v) Low fiber
(vi) Low *cis*-unsaturated fats
(vii) High refined carbohydrates
Life style
(i) Low grade physical activity
(ii) Prolonged fasting
(iii) Rapid weight loss
(iv) Pregnancy and parity
(v) Oral contraceptives
(vi) Estrogen replacement therapy
Associated conditions
(i) The metabolic syndrome
(ii) Obesity
(iii) Insulin resistance
(iv) Diabetes type 2
(v) Nonalcoholic fatty liver disease
(vi) Gallbladder and/or intestinal stasis

Adapted and modified from Portincasa et al. The Lancet, 2006 [17].

## Abbreviations

ABC: ATP-binding cassette (transporter)
NPC1L1: Niemann-Pick C1-like 1 protein. Adapted from de Bari et al. [130], Wang et al. [21], and Portincasa and Wang [6].

## References

[1] Everhart JE, Ruhl CE (2009). Burden of digestive diseases in the united states part I: overall and upper gastrointestinal diseases. *Gastroenterology*.

[2] Shaffer EA (2005). Epidemiology and risk factors for gallstone disease: has the paradigm changed in the 21st century?. *Current Gastroenterology Reports*.

[3] Attili AF, Capocaccia R, Carulli N (1997). Factors associated with gallstone disease in the MICOL experience. *Hepatology*.

[4] Attili AF, Carulli N, Roda E (1995). Epidemiology of gallstone disease in Italy: prevalence data of the multicenter Italian study on cholelithiasis (M.I.COL.). *The American Journal of Epidemiology*.

[5] Diehl AK (1991). Epidemiology and natural history of gallstone disease. *Gastroenterology Clinics of North America*.

[6] Portincasa P, Wang DQ (2012). Intestinal absorption, hepatic synthesis, and biliary secretion of cholesterol: where are we for cholesterol gallstone formation?. *Hepatology*.

[7] Eckel RH, Grundy SM, Zimmet PZ (2005). The metabolic syndrome. *The Lancet*.

[8] Grundy SM (2005). Metabolic syndrome scientific statement by the American heart association and the national heart, lung, and blood institute. *Arteriosclerosis, Thrombosis, and Vascular Biology*.

[9] Grundy SM, Barnett JP (1990). Metabolic and health complications of obesity. *Disease-a-Month*.

[10] Grundy SM, Cleeman JI, Daniels SR (2005). Diagnosis and management of the metabolic syndrome: an American heart association/national heart, lung, and blood institute scientific statement. *Circulation*.

[11] Tsai CJ, Leitzmann MF, Willett WC, Giovannucci EL (2004). Prospective study of abdominal adiposity and gallstone disease in US men. *The American Journal of Clinical Nutrition*.

[12] Méndez-Sánchez N, Chavez-Tapia NC, Motola-Kuba D (2005). Metabolic syndrome as a risk factor for gallstone disease. *World Journal of Gastroenterology*.

[13] Nervi F, Miquel JF, Alvarez M (2006). Gallbladder disease is associated with insulin resistance in a high risk Hispanic population. *Journal of Hepatology*.

[14] Ata N, Kucukazman M, Yavuz B (2011). The metabolic syndrome is associated with complicated gallstone disease. *Canadian Journal of Gastroenterology*.

[15] Wang DQ, Cohen DE, Carey MC (2009). Biliary lipids and cholesterol gallstone disease. *Journal of Lipid Research*.

[16] Mazzio EA, Soliman KF (2012). Basic concepts of epigenetics: impact of environmental signals on gene expression. *Epigenetics*.

[17] Portincasa P, Moschetta A, Palasciano G (2006). Cholesterol gallstone disease. *The Lancet*.

[18] Hofmann AF, Amelsberg A, Vansonnenberg E (1993). Pathogenesis and treatment of gallstones. *The New England Journal of Medicine*.

[19] Lamont JT, Carey MC (1992). Cholesterol gallstone formation. 2. Pathobiology and pathomechanics. *Progress in liver diseases*.

[20] Portincasa P, Di Ciaula A, Baldassarre G (1994). Gallbladder motel function in gallstone patients: sonographic and in vitro studies on the role of gallstones; smooth muscle function and gallbladder wall inflammation. *Journal of Hepatology*.

[21] Wang DQ, Neuschwander-Tetri BA, Portincasa P (2012). *The Biliary System*.

[22] Lammert F, Miquel JF (2008). Gallstone disease: from genes to evidence-based therapy. *Journal of Hepatology*.

[23] Lammert F, Sauerbruch T (2005). Mechanisms of disease: the genetic epidemiology of gallbladder stones. *Nature Clinical Practice Gastroenterology and Hepatology*.

[24] Portincasa P, Di Ciaula A, Wang HH (2008). Coordinate regulation of gallbladder motor function in the gut-liver axis. *Hepatology*.

[25] Portincasa P, Moschetta A, Puglisi F, Wang DQH, Borzellino G, Cordiano C (2008). Medical treatment of gallstone disease. *Biliary Lithiasis: Basic Science, Current Diagnosis and Management*.

[26] Wang DQH, Zhang L, Wang HH (2005). High cholesterol absorption efficiency and rapid biliary secretion of chylomicron remnant cholesterol enhance cholelithogenesis in gallstone-susceptible mice. *Biochimica et Biophysica Acta*.

[27] Wang HH, Portincasa P, Wang DQH (2008). Molecular pathophysiology and physical chemistry of cholesterol gallstones. *Frontiers in Bioscience*.

[28] Wittenburg H, Lammert F (2007). Genetic predisposition to gallbladder stones. *Seminars in Liver Disease*.

[29] Wang DQH, Afdhal NH, Feldman M, Friedman LS, Brand LJ (2010). Gallstone disease. *Sleisenger and Fordtran's Gastrointestinal and Liver Disease*.

[30] Portincasa P, Calamita G (2012). Water channel proteins in bile formation and flow in health and disease: when immiscible becomes miscible. *Molecular Aspects of Medicine*.

[31] Admirand WH, Small DM (1968). The physicochemical basis of cholesterol gallstone formation in man. *Journal of Clinical Investigation*.

[32] Wang DQH, Carey MC (1996). Characterization of crystallization pathways during cholesterol precipitation from human gallbladder biles: identical pathways to corresponding model biles with three predominating sequences. *Journal of Lipid Research*.

[33] Clarenbach JJ, Reber M, Lütjohann D, von Bergmann K, Sudhop T (2006). The lipid-lowering effect of ezetimibe in pure vegetarians. *Journal of Lipid Research*.

[34] Kozarsky KF, Donahee MH, Rigotti A, Iqbal SN, Edelman ER, Krieger M (1997). Overexpression of the HDL receptor SR-BI alters plasma HDL and bile cholesterol levels. *Nature*.

[35] Robins SJ, Fasulo JM (1997). High density lipoproteins, but not other lipoproteins, provide a vehicle for sterol transport to bile. *Journal of Clinical Investigation*.

[36] Sehayek E, Ono JG, Shefer S (1998). Biliary cholesterol excretion: a novel mechanism that regulates dietary cholesterol absorption. *Proceedings of the National Academy of Sciences of the United States of America*.

[37] Oram JF, Heinecke JW (2005). ATP-binding cassette transporter A1: a cell cholesterol exporter that protects against cardiovascular disease. *Physiological Reviews*.

[38] Kesaniemi YA, Miettinen TA (1987). Cholesterol absorption efficiency regulates plasma cholesterol level in the Finnish population. *European Journal of Clinical Investigation*.

[39] Bosner MS, Lange LG, Stenson WF, Ostlund RE (1999). Percent cholesterol absorption in normal women and men quantified with dual stable isotopic tracers and negative ion mass spectrometry. *Journal of Lipid Research*.

[40] Altmann SW, Davis HR, Zhu LJ (2004). Niemann-pick C1 like 1 protein is critical for intestinal cholesterol absorption. *Science*.

[41] Wang DQH (2007). Regulation of intestinal cholesterol absorption. *Annual Review of Physiology*.

[42] Wang DQH, Lee SP (2008). Physical chemistry of intestinal absorption of biliary cholesterol in mice. *Hepatology*.

[43] Turley SD, Dietschy JM, Arias IM, Iakoby WB, Popper H, Schachter D, Shafritz DA (1988). The metabolism and excretion of cholesterol by the liver. *The Liver: Biology and Pathobiology*.

[44] Long MA, Kaler EW, Lee SP (1994). Structural characterization of the micelle-vesicle transition in lecithin-bile salt solutions. *Biophysical Journal*.

[45] Quarfordt SH, Oswald B, Landis B, Xu HS, Zhang SH, Maeda N (1995). In vivo cholesterol kinetics in apolipoprotein E-deficient and control mice. *Journal of Lipid Research*.

[46] Robins SJ, Brunengraber H (1982). Origin of biliary cholesterol and lecithin in the rat: contribution of new synthesis and preformed hepatic stores. *Journal of Lipid Research*.

[47] Schwartz CC, Berman M, Vlahcevic ZR (1978). Multicompartmental analysis of cholesterol metabolism in man. Characterization of the hepatic bile acid and biliary cholesterol precursor sites. *Journal of Clinical Investigation*.

[48] Chen Q, Li WJ, Wan YY, Yu CD, Li WG (2012). Fibroblast growth factor receptor 4 Gly388Arg polymorphism associated with severity of gallstone disease in a Chinese population. *Genetics and Molecular Research*.

[49] Beaven SW, Tontonoz P (2006). Nuclear receptors in lipid metabolism: targeting the heart of dyslipidemia. *Annual Review of Medicine*.

[50] Peet DJ, Janowski BA, Mangelsdorf DJ (1998). The LXRs: a new class of oxysterol receptors. *Current Opinion in Genetics and Development*.

[51] Tontonoz P, Mangelsdorf DJ (2003). Liver X receptor signaling pathways in cardiovascular disease. *Molecular Endocrinology*.

[52] Uppal H, Zhai Y, Gangopadhyay A (2008). Activation of liver X receptor sensitizes mice to gallbladder cholesterol crystallization. *Hepatology*.

[53] Tsai CJ, Leitzmann MF, Willett WC, Giovannucci EL (2009). Statin use and the risk of cholecystectomy in women. *Gastroenterology*.

[54] Erichsen R, Frøslev T, Lash TL, Pedersen L, Sørensen HT (2011). Long-term statin use and the risk of gallstone disease: a population-based case-control study. *The American Journal of Epidemiology*.

[55] Chapman BA, Burt MJ, Chisholm RJ, Allan RB, Yeo KHJ, Ross AG (1998). Dissolution of gallstones with simvastatin, an HMG CoA reductase inhibitor. *Digestive Diseases and Sciences*.

[56] Hanson DS, Duane WC (1994). Effects of lovastatin and chenodiol on bile acid synthesis, bile lipid composition, and biliary lipid secretion in healthy human subjects. *Journal of Lipid Research*.

[57] Smit JWA, van Erpecum KJ, Portincasa P, Renooij W, Erkelens DW, van Berge-Henegouwen GP (1995). Effects of simvastatin and cholestyramine on bile lipid composition and gall bladder motility in patients with hypercholesterolaemia. *Gut*.

[58] Mitchell JC, Logan GM, Stone BG, Duane WC (1991). Effects of lovastatin on biliary lipid secretion and bile acid metabolism in humans. *Journal of Lipid Research*.

[59] Smith JL, Roach PD, Wittenberg LN (2000). Effects of simvastatin on hepatic cholesterol metabolism, bile lithogenicity and bile acid hydrophobicity in patients with gallstones. *Journal of Gastroenterology and Hepatology*.

[60] Vanhanen H, Kesaniemi YA, Miettinen TA (1992). Pravastatin lowers serum cholesterol, cholesterol-precursor sterols, fecal steroids, and cholesterol absorption in man. *Metabolism: Clinical and Experimental*.

[61] Lammert F, Wang DQH, Paigen B, Carey MC (1999). Phenotypic characterization of Lith genes that determine susceptibility to cholesterol cholelithiasis in inbred mice: integrated activities of hepatic lipid regulatory enzymes. *Journal of Lipid Research*.

[62] Grundy SM, Metzger AL, Adler RD (1972). Mechanisms of lithogenic bile formation in American Indian women with cholesterol gallstones. *Journal of Clinical Investigation*.

[63] Key PH, Bonorris GG, Marks JW (1980). Biliary lipid synthesis and secretion in gallstone patients before and during treatment with chenodeoxycholic acid. *Journal of Laboratory and Clinical Medicine*.

[64] Nervi FO, Covarrubias CF, Valdivieso VD, Ronco BO, Solari A, Tocornal J (1981). Hepatic cholesterogenesis in Chileans with cholesterol gallstone disease. Evidence for sex differences in the regulation of hepatic cholesterol metabolism. *Gastroenterology*.

[65] Salen G, Nicolau G, Shefer S, Mosbach EH (1975). Hepatic cholesterol metabolism in patients with gallstones. *Gastroenterology*.

[66] Stange EF, Dietschy JM, Danielsson H, Sjovall J (1985). Cholesterol absorption and metabolism by the intestinal epithelium. *Sterols and Bile Acids*.

[67] Mok HYI, von Bergmann K, Grundy SM (1979). Effects of continuous and intermittent feeding on biliary lipid outputs in man: application for measurements of intestinal absorption of cholesterol and bile acids. *Journal of Lipid Research*.

[68] Buhman KK, Accad M, Novak S (2000). Resistance to diet-induced hypercholesterolemia and gallstone formation in ACAT2-deficient mice. *Nature Medicine*.

[69] Wang HH, Wang DQH (2005). Reduced susceptibility to cholesterol gallstone formation in mice that do not produce apolipoprotein B48 in the intestine. *Hepatology*.

[70] Amigo L, Quiones V, Mardones P (2000). Impaired biliary cholesterol secretion and decreased gallstone formation in apolipoprotein E-deficient mice fed a high-cholesterol diet. *Gastroenterology*.

[71] DenBesten L, Connor WE, Bell S (1973). The effect of dietary cholesterol on the composition of human bile. *Surgery*.

[72] Dam H, Prange I, Jensen MK, Kallehauge HE, Fenger HJ (1971). Studies on human bile—IV. Influence of ingestion of cholesterol in the form of eggs on the composition of bile in healthy subjects. *Zeitschrift für Ernährungswissenschaft*.

[73] Andersen E, Hellstrom K (1979). The effect of cholesterol feeding on bile acid kinetics and biliary lipids in normolipidemic and hypertriglyceridemic subjects. *Journal of Lipid Research*.

[74] Lee DWT, Gilmore CJ, Bonorris G (1985). Effect of dietary cholesterol on biliary lipids in patients with gallstones and normal subjects. *The American Journal of Clinical Nutrition*.

[75] Gilat T, Feldman C, Halpern Z (1983). An increased familial frequency of gallstones. *Gastroenterology*.

[76] Sampliner RE, Bennett PH, Comess LJ, Rose FA, Burch TA (1970). Gallbladder disease in pima indians. Demonstration of high prevalence and early onset by cholecystography. *The New England Journal of Medicine*.

[77] Sarin SK, Negi VS, Dewan R, Sasan S, Saraya A (1995). High familial prevalence of gallstones in the first-degree relatives of gallstone patients. *Hepatology*.

[78] Stokes CS, Krawczyk M, Lammert F (2011). Gallstones: environment, lifestyle and genes. *Digestive Diseases*.

[79] Wittenburg H (2010). Hereditary liver disease: gallstones. *Best Practice and Research: Clinical Gastroenterology*.

[80] Katsika D, Grjibovski A, Einarsson C, Lammert F, Lichtenstein P, Marschall HU (2005). Genetic and environmental influences on symptomatic gallstone disease: a Swedish study of 43,141 twin pairs. *Hepatology*.

[81] Katsika D, Magnusson P, Krawczyk M (2010). Gallstone disease in Swedish twins: risk is associated with ABCG8 D19H genotype. *Journal of Internal Medicine*.

[82] Acalovschi M, Ciocan A, Mostean O (2006). Are plasma lipid levels related to ABCG5/ABCG8 polymorphisms? A preliminary study in siblings with gallstones. *European Journal of Internal Medicine*.

[83] Gylling H, Hallikainen M, Pihlajamäki J (2004). Polymorphisms in the ABCG5 and ABCG8 genes associate with cholesterol absorption and insulin sensitivity. *Journal of Lipid Research*.

[84] Chuang SC, Hsi E, Lee KT (2012). Mucin genes in gallstone disease. *Clinica Chimica Acta*.

[85] Chuang SC, Hsi E, Wang SN, Yu ML, Lee KT, Juo SH (2011). Polymorphism at the mucin-like protocadherin gene influences susceptibility to gallstone disease. *Clinica Chimica Acta*.

[86] Xu HL, Cheng JR, Andreotti G (2011). Cholesterol metabolism gene polymorphisms and the risk of biliary tract cancers and stones: a population-based case-control study in Shanghai, China. *Carcinogenesis*.

[87] Berends LM, Ozanne SE (2012). Early determinants of type-2 diabetes. *Best Practice and Research Clinical Endocrinology and Metabolism*.

[88] Gilbert ER, Liu D (2012). Epigenetics: the missing link to understanding beta-cell dysfunction in the pathogenesis of type 2 diabetes. *Epigenetics*.

[89] Martin-Gronert MS, Ozanne SE (2012). Metabolic programming of insulin action and secretion. *Diabetes, Obesity and Metabolism*.

[90] Milagro FI, Mansego ML, De MC, Martinez JA (2012). Dietary factors, epigenetic modifications and obesity outcomes: progresses and perspectives. *Molecular Aspects of Medicine*.

[91] Sookoian S, Pirola CJ (2012). DNA methylation and hepatic insulin resistance and steatosis. *Current Opinion in Clinical Nutrition and Metabolic Care*.

[92] Wang J, Wu Z, Li D (2012). Nutrition, epigenetics, and metabolic syndrome. *Antioxidants and Redox Signaling*.

[93] Dawson MA, Kouzarides T (2012). Cancer epigenetics: from mechanism to therapy. *Cell*.

[94] Dawson MA, Kouzarides T, Huntly BJ (2012). Targeting epigenetic readers in cancer. *The New England Journal of Medicine*.

[95] Marsit C, Christensen B (2013). Blood-derived DNA methylation markers of cancer risk. *Advances in Experimental Medicine and Biology*.

[96] Udali S, Guarini P, Moruzzi S, Choi SW, Friso S (2012). Cardiovascular epigenetics: from DNA methylation to microRNAs. *Molecular Aspects of Medicine*.

[97] Kwok JB (2010). Role of epigenetics in Alzheimers and Parkinsons disease. *Epigenomics*.

[98] Piedrahita JA (2011). The role of imprinted genes in fetal growth abnormalities. *Birth Defects Research A*.

[99] Loke YJ, Novakovic B, Ollikainen M (2013). The peri/postnatal epigenetic twins study (PETS). *Twin Research and Human Genetics*.

[100] Low FM, Gluckman PD, Hanson MA (2011). Developmental plasticity and epigenetic mechanisms underpinning metabolic and cardiovascular diseases. *Epigenomics*.

[101] Cong R, Jia Y, Li R (2012). Maternal low-protein diet causes epigenetic deregulation of HMGCR and CYP7alpha1 in the liver of weaning piglets. *The Journal of Nutritional Biochemistry*.

[102] Sohi G, Marchand K, Revesz A, Arany E, Hardy DB (2011). Maternal protein restriction elevates cholesterol in adult rat offspring due to repressive changes in histone modifications at the cholesterol 7*α*-hydroxylase promoter. *Molecular Endocrinology*.

[103] Cheng TF, Choudhuri S, Muldoon-Jacobs K (2012). Epigenetic targets of some toxicologically relevant metals: a review of the literature. *Journal of Applied Toxicology*.

[104] Martinez-Zamudio R, Ha HC (2011). Environmental epigenetics in metal exposure. *Epigenetics*.

[105] Reichard JF, Puga A (2010). Effects of arsenic exposure on DNA methylation and epigenetic gene regulation. *Epigenomics*.

[106] Sadli N, Ackland ML, De MD, Sinclair AJ, Suphioglu C (2012). Effects of zinc and DHA on the epigenetic regulation of human neuronal cells. *Cellular Physiology and Biochemistry*.

[107] Wang B, Li Y, Shao C, Tan Y, Cai L (2012). Cadmium and its epigenetic effects. *Current Medicinal Chemistry*.

[108] Ding T, McConaha M, Boyd KL, Osteen KG, Bruner-Tran KL (2011). Developmental dioxin exposure of either parent is associated with an increased risk of preterm birth in adult mice. *Reproductive Toxicology*.

[109] McKinlay R, Plant JA, Bell JNB, Voulvoulis N (2008). Calculating human exposure to endocrine disrupting pesticides via agricultural and non-agricultural exposure routes. *Science of the Total Environment*.

[110] Nilsson E, Larsen G, Manikkam M, Guerrero-Bosagna C, Savenkova MI, Skinner MK (2012). Environmentally induced epigenetic transgenerational inheritance of ovarian disease. *PloS ONE*.

[111] Takeda T, Fujii M, Taura J, Ishii Y, Yamada H (2012). Dioxin silences gonadotropin expression in perinatal pups by inducing histone deacetylases: a new insight into the mechanism for the imprinting of sexual immaturity by dioxin. *The Journal of Biological Chemistry*.

[112] Weinhold B (2012). More chemicals show epigenetic effects across generations. *Environmental Health Perspectives*.

[113] Ferrari A, Fiorino E, Giudici M (2012). Linking epigenetics to lipid metabolism: focus on histone deacetylases. *Molecular Membrane Biology*.

[114] Chuang JC, Jones PA (2007). Epigenetics and microRNAs. *Pediatric Research*.

[115] Tang WY, Ho SM (2007). Epigenetic reprogramming and imprinting in origins of disease. *Reviews in Endocrine and Metabolic Disorders*.

[116] Martinez JA, Cordero P, Campion J, Milagro FI (2012). Interplay of early-life nutritional programming on obesity, inflammation and epigenetic outcomes. *The Proceedings of the Nutrition Society*.

[117] Rhee SY, Hwang YC, Woo JT (2013). Blood lead is significantly associated with metabolic syndrome in Korean adults: an analysis based on the Korea national health and nutrition examination survey (KNHANES), 2008. *Cardiovascular Diabetology*.

[118] Unisa S, Jagannath P, Dhir V, Khandelwal C, Sarangi L, Roy TK (2011). Population-based study to estimate prevalence and determine risk factors of gallbladder diseases in the rural Gangetic basin of North India. *HPB*.

[119] Ambros V (2004). The functions of animal microRNAs. *Nature*.

[120] Bartel DP (2004). MicroRNAs: genomics, biogenesis, mechanism, and function. *Cell*.

[121] Bartel DP (2009). MicroRNAs: target recognition and regulatory functions. *Cell*.

[122] Moore KJ, Rayner KJ, Suárez Y, Fernández-Hernando C (2010). MicroRNAs and cholesterol metabolism. *Trends in Endocrinology and Metabolism*.

[123] Ross MG, Beall MH (2008). Adult sequelae of intrauterine growth restriction. *Seminars in Perinatology*.

[124] Lamarche B, Lemieux S, Dagenais GR, Després JP (1998). Visceral obesity and the risk of ischaemic heart disease: insights from the Québec cardiovascular study. *Growth Hormone and IGF Research*.

[125] Makishima M, Okamoto AY, Repa JJ (1999). Identification of a nuclear receptor for bite acids. *Science*.

[126] Parks DJ, Blanchard SG, Bledsoe RK (1999). Bile acids: natural ligands for an orphan nuclear receptor. *Science*.

[127] Repa JJ, Mangelsdorf DJ (2002). The liver X receptor gene team: potential new players in atherosclerosis. *Nature Medicine*.

[128] Kalaany NY, Mangelsdorf DJ (2006). LXRs and FXR: the Yin and Yang of cholesterol and fat metabolism. *Annual Review of Physiology*.

[129] Lee J, Seok S, Yu P (2012). Genomic analysis of hepatic farnesoid X receptor binding sites reveals altered binding in obesity and direct gene repression by farnesoid X receptor in mice. *Hepatology*.

[130] de Bari O, Neuschwander-Tetri BA, Liu M, Portincasa P, Wang DQ (2012). Ezetimibe: its novel effects on the prevention and the treatment of cholesterol gallstones and nonalcoholic fatty liver disease. *Journal of Lipids*.

